# Concordance among experts in assessing apical mucosal preservation during holmium laser enucleation of the prostate (HoLEP): implications for artificial intelligence model development

**DOI:** 10.1007/s00345-025-06118-x

**Published:** 2025-12-04

**Authors:** Archan Khandekar, Aravindh Rathinam, Ansh Bhatia, Diana M. Lopategui, Jonathan Katz, Roger L. Sur, Nicholas Smith, Pankaj N. Maheshwari, Hemendra N. Shah

**Affiliations:** 1 Desai Sethi Urology Institute, Miller School of Medicine, University of Miami, 1120 NW 14th St #2107, 15th Floor, Miami, FL 33136 USA; 2 Department of Interventional Radiology, University of Miami, Miami, FL USA; 3 Department of Urology, University of California, San Diego, CA USA; 4 Mount Sinai Medical Center, Miami, FL USA; 5 Fortis Hospital, Mumbai, India

**Keywords:** Prostatic hyperplasia, Holmium, Lasers, Surgical, Urinary incontinence, Stress, Computer vision, Reproducibility of results

## Abstract

**Objective:**

To quantify interrater reliability among expert urologists in visually assessing apical mucosal preservation during holmium laser enucleation of the prostate (HoLEP) and to examine the association between preservation ratings and early postoperative continence, thereby informing the design of computer‑vision algorithms.

**Methods:**

Sixty anonymized video segments from “en‑bloc” HoLEP procedures performed between June 2023 and May 2024 were independently reviewed by six HoLEP surgeons. Each rater classified mucosal integrity as completely preserved, partially preserved, or not preserved. Interrater agreement was quantified with pairwise Cohen’s κ and multi‑rater Fleiss κ. Predictive value for 6‑week continence was evaluated using logistic regression and receiver‑operating‑characteristic (ROC) analysis of consensus ratings.

**Results:**

Pairwise Cohen’s κ ranged from 0.07 to 0.44; overall Fleiss κ was 0.18, indicating poor concordance. Agreement was highest between surgeons trained at the same institution (κ 0.44). The partially preserved class accounted for most disagreements. Majority‑vote preservation grade predicted continence poorly (ROC‑AUC 0.60). Observed patterns suggested that worse mucosal preservation tended to coincide with higher incontinence rates, but these trends were exploratory and not suitable for inferential interpretation.

**Conclusion:**

Expert visual assessment of apical mucosal preservation lacks sufficient reliability to serve as ground truth for supervised computer‑vision training. Given the non-convergent regression model and exploratory nature of observed associations, mucosal-preservation ratings should not be used as inferential predictors. Standardized grading criteria or outcome‑based labels are needed to develop robust AI tools aimed at reducing transient stress urinary incontinence after HoLEP.

**Supplementary Information:**

The online version contains supplementary material available at 10.1007/s00345-025-06118-x.

## Introduction

Holmium laser enucleation of the prostate (HoLEP) is a well-established surgical treatment for benign prostatic hyperplasia (BPH) [1]. Transient stress urinary incontinence (TSUI) is a common consequence of HoLEP, with reported rates of up to 44% in the early postoperative period [2]. However, contemporary rates have decreased substantially with the adoption of technical modifications such as early apical release and improved mucosal preservation techniques [3–5]. Recent randomized trial evidence comparing en-bloc and lobe-by-lobe techniques further highlights how surgical technique influences early continence outcomes [6].

In this context, computer vision technologies that enable the automated assessment of apical mucosa preservation based on procedural videos could have significant prognostic value and guide intraoperative or postoperative care. Artificial intelligence (AI) algorithms are already extracting similar intraoperative information successfully, such as surgical steps, safety views, and event durations [7, 8] in a range of specialties and procedure types. Within urology, AI has been applied to tasks such as surgical phase recognition in robotic prostatectomy and automated Gleason grading [9, 10]. However, a significant gap remains in the development of validated AI tools that can analyze intraoperative events to predict postoperative *functional outcomes*, such as urinary continence. Such algorithms are often developed using supervised learning, with clinical domain experts labeling the intraoperative events to define the ground truth. In this process, algorithm utility, accuracy, and generalizability depend on well-defined and reliable criteria, as models trained on noisy, conflicting labels can lead to unpredictable and erroneous predictions [11].

Evaluating concordance between expert clinicians in visually classifying apical mucosa preservation is therefore a crucial first step in assessing the feasibility of AI-based classification during HoLEP. In this study, we aimed to determine how consistently expert urologists from different institutions rate apical mucosal preservation in HoLEP videos, as a potential basis for training a computer vision model. We also assessed associations between their ratings and postoperative incontinence. Considering the findings, we explored strategies to improve annotation reliability and adapt our AI modeling approach.

## Methods

We retrospectively reviewed data of all the men who underwent "en-bloc" HoLEP at our institution between June 23 and May 2024 to identify those patients whose surgical video recordings were available for review. The surgical technique was a standardized 'en-bloc' method without early apical release [12]. Briefly, all procedures were performed or supervised by a single urologist with over 20 years of experience using a (100 W) holmium laser machine or its Moses pulse modulation (MOSES™ 1.0 or MOSES™ 2.0) and a 550-μm laser fibre (Boston Scientific, Marlborough, MA, USA). Laser settings for enucleation were standardized across all cases at 2 J and 30 Hz. Morcellation was performed with a Versacut or PIRANHA morcellator (Richard Wolf, Chicago, IL, USA). The procedure was performed or supervised by a single surgeon, with active participation from residents and endourology fellows. Data of these patient’s was reviewed to identify those with associated urethral strictures, neurogenic bladder, bladder tumors and those with history of pelvic radiation and these patients were excluded from study. Patients with locally advanced prostate cancer undergoing channel procedures were also excluded. Patients with pre-existing urinary incontinence and those lacking continence outcome at 6 weeks were also excluded. A total of 60 consecutive intraoperative video sequences met predefined legibility and signal-to-noise thresholds, rendering them suitable for downstream processing and feature extraction by the computer vision pipeline. No videos were excluded due to poor image resolution. This database was approved by our Institutional Review Board (IRB no: 20180511).

High-quality surgical video and continence outcomes were available for all included cases. For each case, we used the Theator Inc. Surgical Intelligence platform (PaltoAlto, USA) to extract an intraoperative video segment capturing the appearance of apical mucosa after completion of enucleation. The clips were anonymized and randomized to mitigate observer bias. We used a dichotomous measure for postoperative urinary continence based on patient-reported leakage and pad use at the first follow-up visit, approximately 6 weeks following the procedure. Participants who reported no leakage or pad use were categorized as “not incontinent” and participants who reported any amount of leakage or pad use were categorized as “incontinent.”

## Expert evaluation of mucosal preservation

**Fig. 1:**
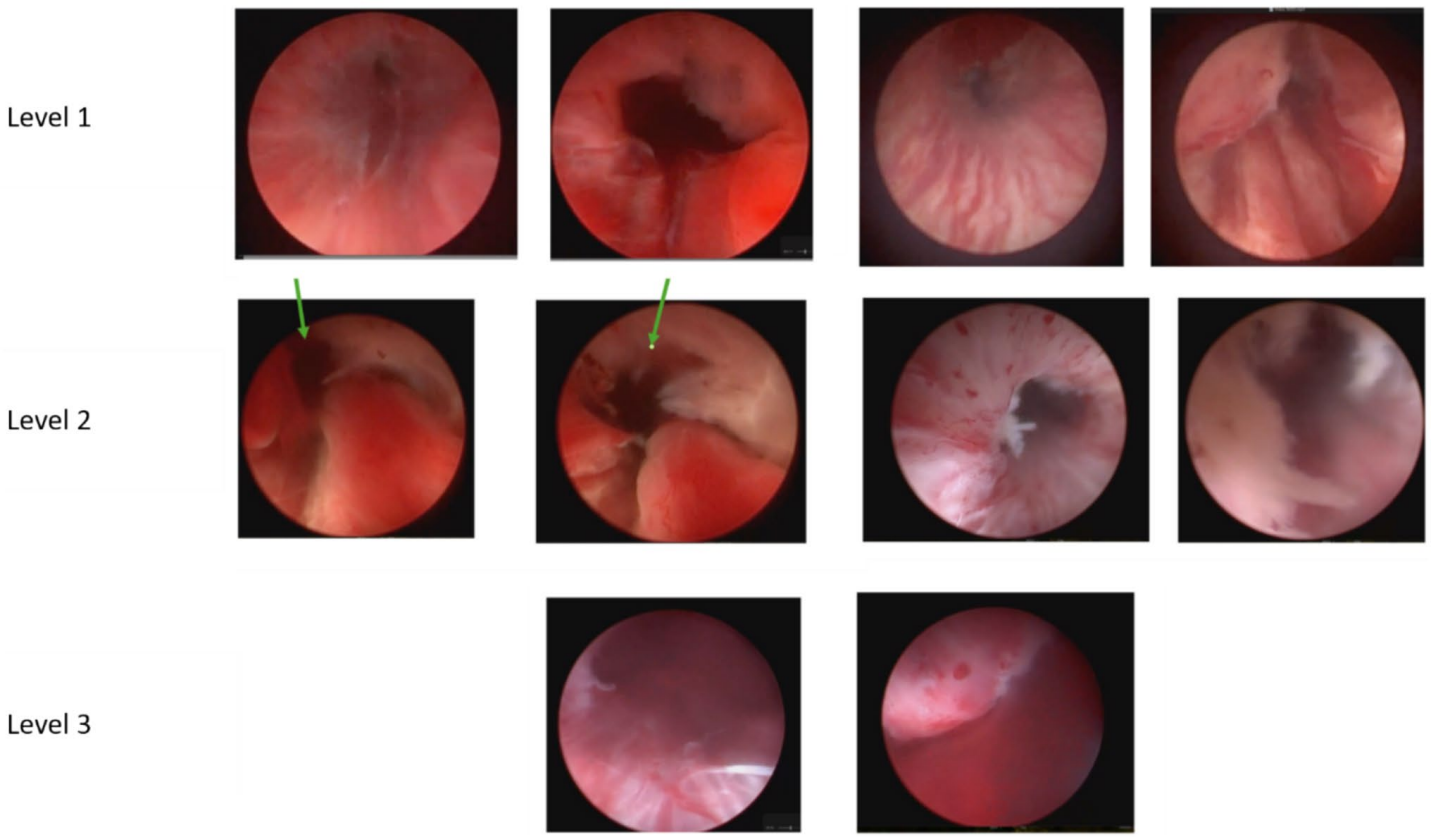
Visual guide for levels of Mucosa preservation. Caption: Visual guide provided to experts for rating mucosa preservation. Level 0: N/A (the mucosa is not visible well enough to assess). Level 1: Mucosa is preserved, the sphincter closes the lumen completely. Level 2: Mucosa has localized defects, the sphincter is closing the urethra well but with partial defects. Level 3: Mucosa is not preserved or has large defects, the lumen is wide open

Six experienced urologists independently reviewed the video clips while blinded to patient identity and continence outcome. Each evaluator classified the degree of apical mucosal preservation using a three-tiered scale: completely preserved—fully intact mucosal lining; partially preserved—partially intact (incompletely ablated) mucosal lining; or not preserved—fully ablated/damaged mucosal lining. Each evaluator was provided with representative pictures showing degree of apical mucosal preservation (Fig. 1).

To capture natural variability in judgment, no formal training was conducted nor were any attempts made to reach consensus.

## Statistical analyses

All statistical analyses were conducted using Rstudio v4.0 (Boston, MA) with appropriate packages for Kappa and Receiver Operating Characteristic (ROC) analysis. A two-sided *p* < 0.05 was considered statistically significant.

*Interrater reliability* For the outcome analysis, a 'consensus-based' rating for each video was determined using a 'majority vote' (i.e., the rating category selected by the most raters). Interrater concordance was evaluated using Cohen’s Kappa (κ) for each pair of evaluators, along with raw percentage agreement for those pairs. We constructed contingency tables for each pair to calculate observed and expected agreement and derived 95% confidence intervals for κ, which we interpreted using standard benchmarks [13]. In addition, we calculated Fleiss’ multi-rater κ [14] to assess overall agreement among the six reviewers.

*Correlation with outcomes* We compared each evaluator’s mucosal preservation rating with the patients' actual continence outcomes. To determine whether the evaluators' consensus ratings could predict postoperative continence, we performed logistic regression and receiver operating characteristic (ROC) analyses. We calculated the area under the curve (AUC), along with sensitivity and specificity, to measure predictive accuracy.

## Results

### Interrater reliability (Fig. 2)

**Fig. 2:**
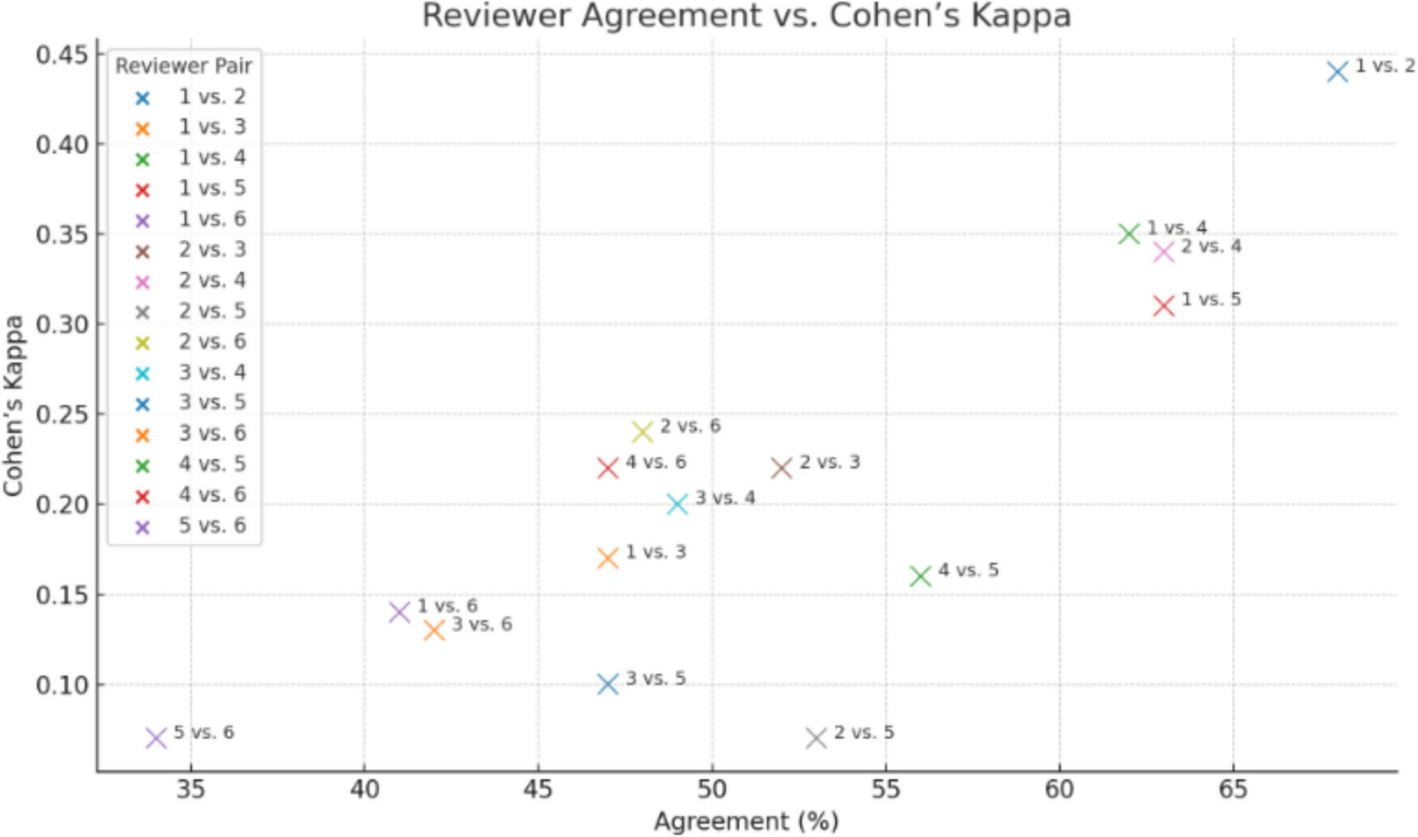
Scatter plot showing reviewer agreement vs Cohen’s kappa

Agreement between the apical mucosa preservation ratings among the six expert reviewers was low to moderate, with Cohen's Kappa ranging from 0.07 to 0.44 across all rater pairs (Tables 1 and 2). The highest agreement was observed between Reviewer 1 and Reviewer 2 (κ = 0.44, 68% agreement), while the lowest was between Reviewer 5 and Reviewer 6 (κ = 0.07, 34% agreement).

**Table 1:** Combined table of ratings

Rater	3-level χ^2^ (df)	*p* (3-level)	2-level χ^2^ (df)	*p* (2-level)	N
Rater 1	5.63 (2)	0.060	5.08 (1)	**0.024**	42
Rater 2	1.84 (2)	0.399	1.79 (1)	0.181	43
Rater 3	2.85 (2)	0.241	2.84 (1)	0.092	47
Rater 4	6.30 (2)	**0.043**	5.84 (1)	**0.016**	46
Rater 5	5.14 (2)	0.076	5.05 (1)	**0.025**	42
Rater 6	0.35 (2)	0.840	0.34 (1)	0.563	40

**Table 2:** Sensitivity analysis showing rating interrater concordance on 2 vs 3 level rating

Rater	% Incontinent by rating (3-level)	3-level *p*	2-level *p*	Direction of association	Sig. (*p* < 0.05)	N
Rater 1	1: 47.6%, 2: 77.8%, 3: 100%	0.060	**0.024**	Higher ratings → More incont.	Yes (2-level)	42
Rater 2	1: 52.0%, 2: 73.3%, 3: 66.7%	0.399	0.181	Weak trend (↑ rating → ↑ incont.)	No	43
Rater 3	1: 50.0%, 2: 75.0%, 3: 73.3%	0.241	0.092	Moderate trend ↑	No	47
Rater 4	1: 43.5%, 2: 82.4%, 3: 66.7%	**0.043**	**0.016**	Strong ↑ rating → ↑ incont.	Yes (both)	46
Rater 5	1: 50.0%, 2: 84.6%, 3: 100%	0.076	**0.025**	Higher ratings → More incont.	Yes (2-level)	42
Rater 6	1: 58.3%, 2: 66.7%, 3: 68.8%	0.840	0.563	Minimal or no trend	No	40

**Fig. 3:**
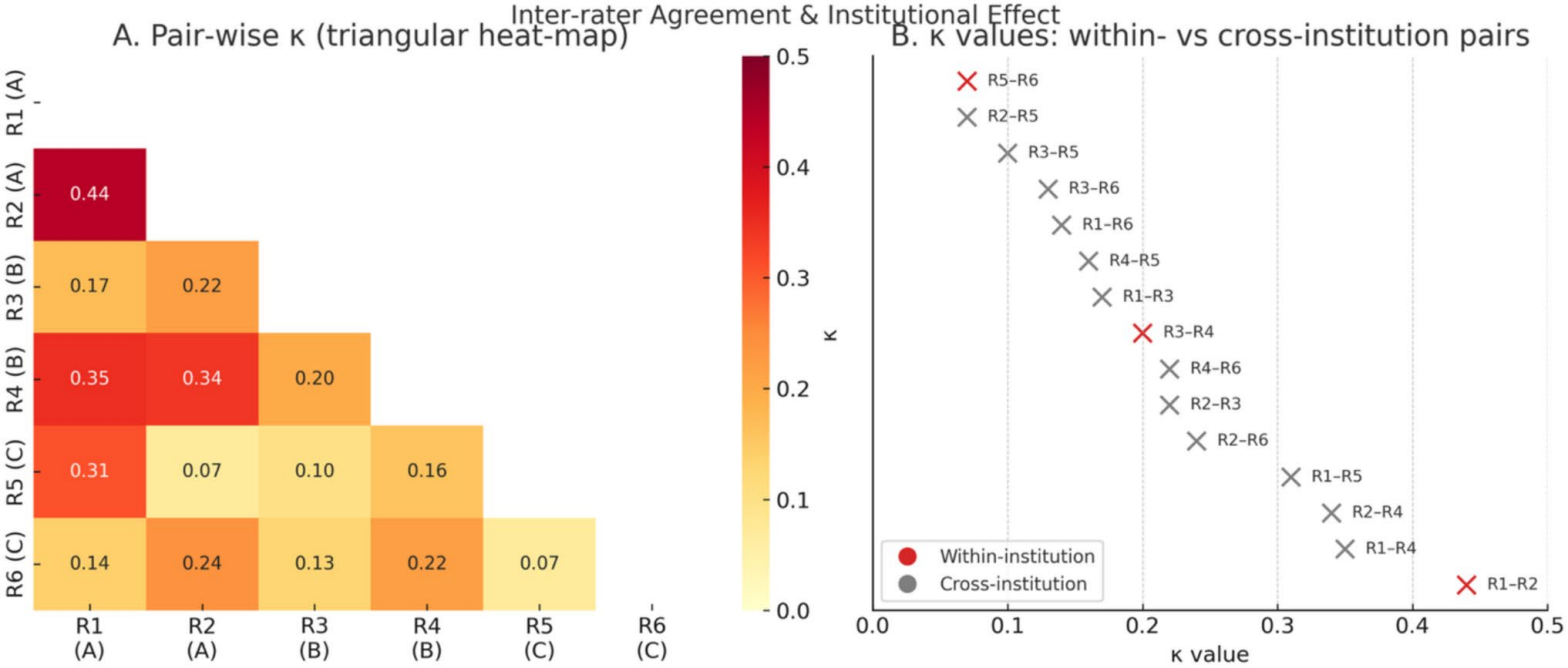
**A** and **B** Interrater agreement subgroup analysis by pairwise match and by institution

Patterns of interrater agreement varied based on the level of mucosal preservation (Fig. 3A). Complete preservation and not preserved cases demonstrated the highest agreement, whereas borderline "partially preserved" cases showed frequent disagreements. Among all cases, only a minority (15%) achieved unanimous ratings, with most cases showing mixed assessments. The Fleiss' Kappa analysis showed an overall κ of 0.18, indicating poor agreement beyond chance when considering all six reviewers simultaneously.

When raters were grouped by training background, R1 and R2 (Fig. 3B), who share the same institutional fellowship and routinely operate together, displayed the highest pair-wise agreement (κ = 0.44) and, on average, agreed more often with each other than with any other colleague. By contrast, κ values between raters trained at different centers rarely exceeded 0.30.

### Correlation with outcomes

In univariate comparisons, mucosal preservation ratings showed a visually observable but weak trend toward higher incontinence with worse preservation categories; however, this association should be interpreted as exploratory rather than inferential. The logistic regression model failed to converge due to quasi-complete separation, rendering regression-based p-values and coefficients non‑interpretable. These results are therefore presented qualitatively rather than inferentially.

Accordingly, we describe the findings qualitatively: worse preservation categories tended to correspond to higher rates of postoperative incontinence for several raters, but these patterns should be regarded as hypothesis‑generating rather than conclusive.

When examining the predictive accuracy using consensus-based (defined by majority vote) mucosal preservation ratings (defined as complete, partial, or no preservation), the receiver operating characteristic (ROC) analysis yielded an area under the curve (AUC) of 0.60, indicating (poor) predictive capability. Thus, using mucosal preservation ratings alone provided only a slight, clinically insignificant improvement over random chance in predicting continence outcomes.

## Discussion

As an initial step toward training a computer vision algorithm to identify the extent of apical mucosa preservation in HoLEP videos, this study assessed the concordance among expert urologists in performing this task. While all evaluators were experienced HoLEP surgeons, their agreement was at best slight to fair when asked to rate the extent of preservation on a 3-level categorical scale.

In practical terms, a Fleiss’ κ of 0.18 signifies 'poor' agreement that is barely above chance, while an AUC of 0.60 represents a 'failing' predictive model. This confirms from a clinical utility perspective that these subjective labels are unsuitable for AI training. Furthermore, while univariate analysis suggested a weak association (*p* = 0.044), this must be interpreted with extreme caution, as the full logistic regression model failed to converge, preventing the calculation of reliable odds ratios or confidence intervals.

Our results lend some support to prior work suggesting that maintaining the integrity of the mucosal lining at the bladder neck and proximal external sphincter during HoLEP can improve postoperative continence outcomes [4, 15]. However, they also suggest that expert preservation assessments are currently too varied to enable accurate evaluation of this relationship, or its effective application in outcome prediction.

This variability, which can likely be attributed to individual thresholds and methods for defining preservation in the absence of distinct visual cues, mirrors other medical domains. Due to pathophysiological, diagnostic and prognostic uncertainties inherent to medicine, ground truths in many clinical areas can be hard to define, even for experts [11]. Aggregated medical data frequently exhibit “noise” stemming from this variability in judgments that would ideally be identical [16].

AI-based interpretation of intraoperative factors could mitigate this human bias and inconsistency [17]. Still, algorithms based on supervised learning reflect the quality of the data used to train them. If labels are inconsistent or noisy, models may struggle to learn meaningful patterns. Alternatively, they might provide an "arbitrarily partial" version of reality, performing in accordance with the labeling patterns of one expert but not another. Indeed, label noise in training data has been shown to result in a range of issues, including decreased classification accuracy, increased number of training samples needed, and difficulties in feature selection [17]. In our results, the lack of strong agreement (with the association between some rater pairs essentially at chance-level) suggested systematic differences in interpretation and judgment. As such, using the labels of any single evaluator as the ground truth would be questionable, and likely result in an algorithm with limited reliability and generalizability.

On one hand, our results indicate that simply asking expert urologists from different institutions and backgrounds to rate HoLEP video footage will not produce a reliable visually based measure of apical mucosa preservation. On the other hand, they suggest that further research and development in this area is worth pursuing, as incontinence outcome predictions for a subset of the raters were encouraging and a majority-based measure showed moderate predictive value. This clustering suggests that local surgical culture and shared mentorship drive a common mental schema for what constitutes “complete,” “partial,” or “no” mucosal preservation. When surgeons are taught the same visual cues and adopt identical thresholds during residency or fellowship, their grading decisions naturally converge; conversely, raters from disparate programs apply subtly different criteria, leading to lower concordance. Such site-specific alignment has important implications: multi-institutional AI models must either harmonise these implicit grading frameworks or explicitly account for institutional label bias to achieve generalisable performance.

Our study significantly builds upon and advances previous research, notably the recent work by Mendelson et al., who evaluated membranous urethral mucosal (MUM) integrity post-HoLEP and its association with urinary incontinence using a limited panel of two experts and two residents in a smaller cohort of 40 patients [18]. While they concluded that MUM visualization alone did not reliably predict postoperative continence outcomes, our study robustly expands upon this initial inquiry by incorporating a larger dataset (60 cases), a broader multi-insitutional international panel of six experienced HoLEP surgeons, and a more comprehensive statistical approach that includes both two-level and three-level analyses and subgroup consensus evaluation. This methodological depth allowed us not only to confirm their observation of limited predictive reliability and substantial inter-observer variability but also to identify specific areas of discrepancy and consensus that highlight potential opportunities for standardized training and clearer visual criteria. Furthermore, by explicitly quantifying outliers and institutional differences, and by integrating advanced statistical techniques such as inter-rater reliability and analysis, our study provides a more detailed roadmap toward refining human annotations and developing reliable AI-based predictive models. Therefore, while Mendelson et al. underscored critical challenges, our work serves as a vital next step by outlining specific pathways forward for standardizing clinical evaluation, enhancing predictive precision, and ultimately improving patient outcomes through more reliable AI-assisted surgical decision-making.

Various approaches could potentially address low agreement between experts regarding preservation to produce algorithms with greater generalizability and predictive power. One direction involves consensus seeking, for example the majority vote method applied in the current study, or having experts discuss and agree upon ground truth labels. While labels reached in this manner could be preferable to single-rater labels, there is evidence that standard consensus seeking methods can also lead to suboptimal models [15]. Another approach would be to maintain multiple independent raters but use agreement or confidence scores to weight cases during training, such that those with higher scores have a greater contribution.

Clearly defined, shared criteria for preserved versus damaged apical mucosa would also be likely to improve consensus between raters, though it would be necessary to validate their association with incontinence outcomes. Heidenberg et al. [15] recently developed a grading scale for external sphincter damage following HoLEP with early apical release. They reported 93% agreement among three raters, as well as a significant association with incontinence measures at three and six months, supporting the possibility that a well-defined classification system could produce sufficiently reliable ground truth ratings for apical mucosa preservation.

Another, fundamentally different approach would be to train an algorithm using actual postoperative incontinence as an objective ground truth. If visual features (presumably related to the state of the apical mucosa) in intraoperative HoLEP videos indeed predict incontinence, this method could produce an accurate algorithm regardless of whether human experts could recognize or agree upon those features.

Finally, the prospective development of AI-guided tools, such as a real-time 'alarm' for sphincter proximity, raises important medico-legal and ethical considerations. Given the inherent anatomical variability, an AI's guidance may not be universally accurate and could create liability if it provides erroneous feedback. Such tools must be rigorously validated and likely implemented as decision-support aids, with the operating surgeon retaining ultimate responsibility and autonomy. Future work must address these frameworks for regulatory approval and safe clinical integration.

## Limitations

Although the reviewer panel was multi-institutional, all HoLEP procedures were performed or supervised by a single lead surgeon at one academic center, potentially limiting the generalizability of findings due to lack of variation in surgical technique. Additionally, while standard instructions and reference images were provided, we did not use a validated grading framework such as the system developed by Heidenberg et al. [15], which may have improved interrater consistency. The use of a three-tiered rating scale that included a “partially preserved” category likely introduced additional subjectivity, as this intermediate classification is inherently less well-defined than a binary “preserved” versus “not preserved” distinction. Additionally, our study was limited by its dichotomous continence definition ('any pad use'), which is less granular than standardized questionnaires and may not capture the full spectrum of TSUI. Our analysis of SUI was also limited to a single intraoperative visual factor. Post-HoLEP continence is multifactorial, and our study did not account for crucial patient-level variables such as age, prostate size, comorbidities, or preoperative catheterization history." Finally, while our sample was sufficient for reliability analysis, our logistic regression model's failure to converge (due to quasi-separation) indicates our study was statistically underpowered for predictive outcome modeling, and no firm conclusions can be drawn from this secondary analysis.

## Conclusion

Given the low interrater reliability and weak predictive power of mucosal preservation ratings alone, an AI-based model trained solely on the annotations of expert urologists would likely have limited accuracy, generalizability, and utility. Alternative approaches to algorithm development, like using standardized classification systems or actual outcomes for training, could be more effective in enabling incontinence prediction based on the extent intraoperative mucosal damage.

**Table:** Interrater agreement table

Reviewer pair	Agreement (%)	Cohen’s kappa
1 vs. 2	68	0.44
1 vs. 3	47	0.17
1 vs. 4	62	0.35
1 vs. 5	63	0.31
1 vs. 6	41	0.14
2 vs. 3	52	0.22
2 vs. 4	63	0.34
2 vs. 5	53	0.07
2 vs. 6	48	0.24
3 vs. 4	49	0.2
3 vs. 5	47	0.1
3 vs. 6	42	0.13
4 vs. 5	56	0.16
4 vs. 6	47	0.22
5 vs. 6	34	0.07

## Supplementary Information

Below is the link to the electronic supplementary material.

Supplementary file1 (DOCX 36 kb)

## Data Availability

No datasets were generated or analysed during the current study.
