# Supplementary material for: Concordance among experts in assessing apical mucosal preservation during holmium laser enucleation of the prostate (HoLEP): implications for artificial intelligence model development
Source: World J Urol. 2025 Dec 4;44(1):20. doi: 10.1007/s00345-025-06118-x (PMC12678557; doi:10.1007/s00345-025-06118-x)
Supplement: Supplementary file 1 — Supplementary file1 (DOCX 36 kb) [file 345_2025_6118_MOESM1_ESM.docx]

**Supplementary material**

**ASSOCIATIONS BETWEEN PRESERVATION RATINGS AND ACTUAL INCONTINENCE STATUS FOR EACH RATER – FOR 3-LEVEL AND 2-LEVEL RATINGS**

**RATER 1**

**3-LEVEL RATING:**

| Contingency Tables | | | | | | | | | |
| --- | --- | --- | --- | --- | --- | --- | --- | --- | --- |
|  | | | | **Incontinence** | | | |  | |
| **1** | |  | | **0** | | **1** | | **Total** | |
| 1 |  | Observed |  | 11 |  | 10 |  | 21 |  |
|  | | % within row |  | 52.4 % |  | 47.6 % |  | 100.0 % |  |
|  | | % within column |  | 73.3 % |  | 37.0 % |  | 50.0 % |  |
| 2 |  | Observed |  | 4 |  | 14 |  | 18 |  |
|  | | % within row |  | 22.2 % |  | 77.8 % |  | 100.0 % |  |
|  | | % within column |  | 26.7 % |  | 51.9 % |  | 42.9 % |  |
| 3 |  | Observed |  | 0 |  | 3 |  | 3 |  |
|  | | % within row |  | 0.0 % |  | 100.0 % |  | 100.0 % |  |
|  | | % within column |  | 0.0 % |  | 11.1 % |  | 7.1 % |  |
| Total |  | Observed |  | 15 |  | 27 |  | 42 |  |
|  | | % within row |  | 35.7 % |  | 64.3 % |  | 100.0 % |  |
|  | | % within column |  | 100.0 % |  | 100.0 % |  | 100.0 % |  |
|  | | | | | | | | | |

| χ² Tests | | | | | | | |
| --- | --- | --- | --- | --- | --- | --- | --- |
|  | | **Value** | | **df** | | **p** | |
| χ² |  | 5.63 |  | 2 |  | 0.060 |  |
| N |  | 42 |  |  | |  | |
|  | | | | | | | |

**2-LEVEL RATING (2 and 3 combined):**

| Contingency Tables | | | | | | | | | |
| --- | --- | --- | --- | --- | --- | --- | --- | --- | --- |
|  | | | | **Incontinence** | | | |  | |
| **12** | |  | | **0** | | **1** | | **Total** | |
| 1 |  | Observed |  | 11 |  | 10 |  | 21 |  |
|  | | % within row |  | 52.4 % |  | 47.6 % |  | 100.0 % |  |
|  | | % within column |  | 73.3 % |  | 37.0 % |  | 50.0 % |  |
| 2 |  | Observed |  | 4 |  | 17 |  | 21 |  |
|  | | % within row |  | 19.0 % |  | 81.0 % |  | 100.0 % |  |
|  | | % within column |  | 26.7 % |  | 63.0 % |  | 50.0 % |  |
| Total |  | Observed |  | 15 |  | 27 |  | 42 |  |
|  | | % within row |  | 35.7 % |  | 64.3 % |  | 100.0 % |  |
|  | | % within column |  | 100.0 % |  | 100.0 % |  | 100.0 % |  |
|  | | | | | | | | | |

| χ² Tests | | | | | | | |
| --- | --- | --- | --- | --- | --- | --- | --- |
|  | | **Value** | | **df** | | **p** | |
| χ² |  | 5.08 |  | 1 |  | 0.024 |  |
| N |  | 42 |  |  | |  | |
|  | | | | | | | |

**RATER 2**

**3-LEVEL RATING:**

| Contingency Tables | | | | | | | | | |
| --- | --- | --- | --- | --- | --- | --- | --- | --- | --- |
|  | | | | **Incontinence** | | | |  | |
| **2** | |  | | **0** | | **1** | | **Total** | |
| 1 |  | Observed |  | 12 |  | 13 |  | 25 |  |
|  | | % within row |  | 48.0 % |  | 52.0 % |  | 100.0 % |  |
|  | | % within column |  | 70.6 % |  | 50.0 % |  | 58.1 % |  |
| 2 |  | Observed |  | 4 |  | 11 |  | 15 |  |
|  | | % within row |  | 26.7 % |  | 73.3 % |  | 100.0 % |  |
|  | | % within column |  | 23.5 % |  | 42.3 % |  | 34.9 % |  |
| 3 |  | Observed |  | 1 |  | 2 |  | 3 |  |
|  | | % within row |  | 33.3 % |  | 66.7 % |  | 100.0 % |  |
|  | | % within column |  | 5.9 % |  | 7.7 % |  | 7.0 % |  |
| Total |  | Observed |  | 17 |  | 26 |  | 43 |  |
|  | | % within row |  | 39.5 % |  | 60.5 % |  | 100.0 % |  |
|  | | % within column |  | 100.0 % |  | 100.0 % |  | 100.0 % |  |
|  | | | | | | | | | |

| χ² Tests | | | | | | | |
| --- | --- | --- | --- | --- | --- | --- | --- |
|  | | **Value** | | **df** | | **p** | |
| χ² |  | 1.84 |  | 2 |  | 0.399 |  |
| N |  | 43 |  |  | |  | |
|  | | | | | | | |

|  |
| --- |

**2-LEVEL RATING (2 and 3 combined):**

| Contingency Tables | | | | | | | | | |
| --- | --- | --- | --- | --- | --- | --- | --- | --- | --- |
|  | | | | **Incontinence** | | | |  | |
| **2** | |  | | **0** | | **1** | | **Total** | |
| 1 |  | Observed |  | 12 |  | 13 |  | 25 |  |
|  | | % within row |  | 48.0 % |  | 52.0 % |  | 100.0 % |  |
|  | | % within column |  | 70.6 % |  | 50.0 % |  | 58.1 % |  |
| 2 |  | Observed |  | 5 |  | 13 |  | 18 |  |
|  | | % within row |  | 27.8 % |  | 72.2 % |  | 100.0 % |  |
|  | | % within column |  | 29.4 % |  | 50.0 % |  | 41.9 % |  |
| Total |  | Observed |  | 17 |  | 26 |  | 43 |  |
|  | | % within row |  | 39.5 % |  | 60.5 % |  | 100.0 % |  |
|  | | % within column |  | 100.0 % |  | 100.0 % |  | 100.0 % |  |
|  | | | | | | | | | |

| χ² Tests | | | | | | | |
| --- | --- | --- | --- | --- | --- | --- | --- |
|  | | **Value** | | **df** | | **p** | |
| χ² |  | 1.79 |  | 1 |  | 0.181 |  |
| N |  | 43 |  |  | |  | |

**RATER 3**

**3-LEVEL RATING:**

| Contingency Tables | | | | | | | | | |
| --- | --- | --- | --- | --- | --- | --- | --- | --- | --- |
|  | | | | **Incontinence** | | | |  | |
| **3** | |  | | **0** | | **1** | | **Total** | |
| 1 |  | Observed |  | 12 |  | 12 |  | 24 |  |
|  | | % within row |  | 50.0 % |  | 50.0 % |  | 100.0 % |  |
|  | | % within column |  | 66.7 % |  | 41.4 % |  | 51.1 % |  |
| 2 |  | Observed |  | 2 |  | 6 |  | 8 |  |
|  | | % within row |  | 25.0 % |  | 75.0 % |  | 100.0 % |  |
|  | | % within column |  | 11.1 % |  | 20.7 % |  | 17.0 % |  |
| 3 |  | Observed |  | 4 |  | 11 |  | 15 |  |
|  | | % within row |  | 26.7 % |  | 73.3 % |  | 100.0 % |  |
|  | | % within column |  | 22.2 % |  | 37.9 % |  | 31.9 % |  |
| Total |  | Observed |  | 18 |  | 29 |  | 47 |  |
|  | | % within row |  | 38.3 % |  | 61.7 % |  | 100.0 % |  |
|  | | % within column |  | 100.0 % |  | 100.0 % |  | 100.0 % |  |
|  | | | | | | | | | |

| χ² Tests | | | | | | | |
| --- | --- | --- | --- | --- | --- | --- | --- |
|  | | **Value** | | **df** | | **p** | |
| χ² |  | 2.85 |  | 2 |  | 0.241 |  |
| N |  | 47 |  |  | |  | |
|  | | | | | | | |

**2-LEVEL RATING (2 and 3 combined):**

| Contingency Tables | | | | | | | | | |
| --- | --- | --- | --- | --- | --- | --- | --- | --- | --- |
|  | | | | **Incontinence** | | | |  | |
| **3** | |  | | **0** | | **1** | | **Total** | |
| 1 |  | Observed |  | 12 |  | 12 |  | 24 |  |
|  | | % within row |  | 50.0 % |  | 50.0 % |  | 100.0 % |  |
|  | | % within column |  | 66.7 % |  | 41.4 % |  | 51.1 % |  |
| 2 |  | Observed |  | 6 |  | 17 |  | 23 |  |
|  | | % within row |  | 26.1 % |  | 73.9 % |  | 100.0 % |  |
|  | | % within column |  | 33.3 % |  | 58.6 % |  | 48.9 % |  |
| Total |  | Observed |  | 18 |  | 29 |  | 47 |  |
|  | | % within row |  | 38.3 % |  | 61.7 % |  | 100.0 % |  |
|  | | % within column |  | 100.0 % |  | 100.0 % |  | 100.0 % |  |
|  | | | | | | | | | |

| χ² Tests | | | | | | | |
| --- | --- | --- | --- | --- | --- | --- | --- |
|  | | **Value** | | **df** | | **p** | |
| χ² |  | 2.84 |  | 1 |  | 0.092 |  |
| N |  | 47 |  |  | |  | |
|  | | | | | | | |

**RATER 4**

**3-LEVEL RATING:**

| Contingency Tables | | | | | | | | | |
| --- | --- | --- | --- | --- | --- | --- | --- | --- | --- |
|  | | | | **Incontinence** | | | |  | |
| **4** | |  | | **0** | | **1** | | **Total** | |
| 1 |  | Observed |  | 13 |  | 10 |  | 23 |  |
|  | | % within row |  | 56.5 % |  | 43.5 % |  | 100.0 % |  |
|  | | % within column |  | 72.2 % |  | 35.7 % |  | 50.0 % |  |
| 2 |  | Observed |  | 3 |  | 14 |  | 17 |  |
|  | | % within row |  | 17.6 % |  | 82.4 % |  | 100.0 % |  |
|  | | % within column |  | 16.7 % |  | 50.0 % |  | 37.0 % |  |
| 3 |  | Observed |  | 2 |  | 4 |  | 6 |  |
|  | | % within row |  | 33.3 % |  | 66.7 % |  | 100.0 % |  |
|  | | % within column |  | 11.1 % |  | 14.3 % |  | 13.0 % |  |
| Total |  | Observed |  | 18 |  | 28 |  | 46 |  |
|  | | % within row |  | 39.1 % |  | 60.9 % |  | 100.0 % |  |
|  | | % within column |  | 100.0 % |  | 100.0 % |  | 100.0 % |  |
|  | | | | | | | | | |

| χ² Tests | | | | | | | |
| --- | --- | --- | --- | --- | --- | --- | --- |
|  | | **Value** | | **df** | | **p** | |
| χ² |  | 6.30 |  | 2 |  | 0.043 |  |
| N |  | 46 |  |  | |  | |
|  | | | | | | | |

**2-LEVEL RATING:**

| Contingency Tables | | | | | | | | | |
| --- | --- | --- | --- | --- | --- | --- | --- | --- | --- |
|  | | | | **Incontinence** | | | |  | |
| **Katz_4dich rating** | |  | | **0** | | **1** | | **Total** | |
| 1 |  | Observed |  | 13 |  | 10 |  | 23 |  |
|  | | % within row |  | 56.5 % |  | 43.5 % |  | 100.0 % |  |
|  | | % within column |  | 72.2 % |  | 35.7 % |  | 50.0 % |  |
| 2 |  | Observed |  | 5 |  | 18 |  | 23 |  |
|  | | % within row |  | 21.7 % |  | 78.3 % |  | 100.0 % |  |
|  | | % within column |  | 27.8 % |  | 64.3 % |  | 50.0 % |  |
| Total |  | Observed |  | 18 |  | 28 |  | 46 |  |
|  | | % within row |  | 39.1 % |  | 60.9 % |  | 100.0 % |  |
|  | | % within column |  | 100.0 % |  | 100.0 % |  | 100.0 % |  |
|  | | | | | | | | | |

| χ² Tests | | | | | | | |
| --- | --- | --- | --- | --- | --- | --- | --- |
|  | | **Value** | | **df** | | **p** | |
| χ² |  | 5.84 |  | 1 |  | 0.016 |  |
| N |  | 46 |  |  | |  | |
|  | | | | | | | |

**RATER 5**

**3-LEVEL RATING:**

| Contingency Tables | | | | | | | | | |
| --- | --- | --- | --- | --- | --- | --- | --- | --- | --- |
|  | | | | **Incontinence** | | | |  | |
| **5** | |  | | **0** | | **1** | | **Total** | |
| 1 |  | Observed |  | 14 |  | 14 |  | 28 |  |
|  | | % within row |  | 50.0 % |  | 50.0 % |  | 100.0 % |  |
|  | | % within column |  | 87.5 % |  | 53.8 % |  | 66.7 % |  |
| 2 |  | Observed |  | 2 |  | 11 |  | 13 |  |
|  | | % within row |  | 15.4 % |  | 84.6 % |  | 100.0 % |  |
|  | | % within column |  | 12.5 % |  | 42.3 % |  | 31.0 % |  |
| 3 |  | Observed |  | 0 |  | 1 |  | 1 |  |
|  | | % within row |  | 0.0 % |  | 100.0 % |  | 100.0 % |  |
|  | | % within column |  | 0.0 % |  | 3.8 % |  | 2.4 % |  |
| Total |  | Observed |  | 16 |  | 26 |  | 42 |  |
|  | | % within row |  | 38.1 % |  | 61.9 % |  | 100.0 % |  |
|  | | % within column |  | 100.0 % |  | 100.0 % |  | 100.0 % |  |
|  | | | | | | | | | |

| χ² Tests | | | | | | | |
| --- | --- | --- | --- | --- | --- | --- | --- |
|  | | **Value** | | **df** | | **p** | |
| χ² |  | 5.14 |  | 2 |  | 0.076 |  |
| N |  | 42 |  |  | |  | |
|  | | | | | | | |

**2-LEVEL RATING:**

| Contingency Tables | | | | | | | | | |
| --- | --- | --- | --- | --- | --- | --- | --- | --- | --- |
|  | | | | **Incontinence** | | | |  | |
| **5** | |  | | **0** | | **1** | | **Total** | |
| 1 |  | Observed |  | 14 |  | 14 |  | 28 |  |
|  | | % within row |  | 50.0 % |  | 50.0 % |  | 100.0 % |  |
|  | | % within column |  | 87.5 % |  | 53.8 % |  | 66.7 % |  |
| 2 |  | Observed |  | 2 |  | 12 |  | 14 |  |
|  | | % within row |  | 14.3 % |  | 85.7 % |  | 100.0 % |  |
|  | | % within column |  | 12.5 % |  | 46.2 % |  | 33.3 % |  |
| Total |  | Observed |  | 16 |  | 26 |  | 42 |  |
|  | | % within row |  | 38.1 % |  | 61.9 % |  | 100.0 % |  |
|  | | % within column |  | 100.0 % |  | 100.0 % |  | 100.0 % |  |
|  | | | | | | | | | |

| χ² Tests | | | | | | | |
| --- | --- | --- | --- | --- | --- | --- | --- |
|  | | **Value** | | **df** | | **p** | |
| χ² |  | 5.05 |  | 1 |  | 0.025 |  |
| N |  | 42 |  |  | |  | |
|  | | | | | | | |

**RATER 6**

**3-LEVEL RATING:**

| Contingency Tables | | | | | | | | | |
| --- | --- | --- | --- | --- | --- | --- | --- | --- | --- |
|  | | | | **Incontinence** | | | |  | |
| **6** | |  | | **0** | | **1** | | **Total** | |
| 1 |  | Observed |  | 5 |  | 7 |  | 12 |  |
|  | | % within row |  | 41.7 % |  | 58.3 % |  | 100.0 % |  |
|  | | % within column |  | 35.7 % |  | 26.9 % |  | 30.0 % |  |
| 2 |  | Observed |  | 4 |  | 8 |  | 12 |  |
|  | | % within row |  | 33.3 % |  | 66.7 % |  | 100.0 % |  |
|  | | % within column |  | 28.6 % |  | 30.8 % |  | 30.0 % |  |
| 3 |  | Observed |  | 5 |  | 11 |  | 16 |  |
|  | | % within row |  | 31.3 % |  | 68.8 % |  | 100.0 % |  |
|  | | % within column |  | 35.7 % |  | 42.3 % |  | 40.0 % |  |
| Total |  | Observed |  | 14 |  | 26 |  | 40 |  |
|  | | % within row |  | 35.0 % |  | 65.0 % |  | 100.0 % |  |
|  | | % within column |  | 100.0 % |  | 100.0 % |  | 100.0 % |  |
|  | | | | | | | | | |

| χ² Tests | | | | | | | |
| --- | --- | --- | --- | --- | --- | --- | --- |
|  | | **Value** | | **df** | | **p** | |
| χ² |  | 0.348 |  | 2 |  | 0.840 |  |
| N |  | 40 |  |  | |  | |
|  | | | | | | | |

**2-LEVEL RATING:**

| Contingency Tables | | | | | | | | | |
| --- | --- | --- | --- | --- | --- | --- | --- | --- | --- |
|  | | | | **Incontinence** | | | |  | |
| **6** | |  | | **0** | | **1** | | **Total** | |
| 1 |  | Observed |  | 5 |  | 7 |  | 12 |  |
|  | | % within row |  | 41.7 % |  | 58.3 % |  | 100.0 % |  |
|  | | % within column |  | 35.7 % |  | 26.9 % |  | 30.0 % |  |
| 2 |  | Observed |  | 9 |  | 19 |  | 28 |  |
|  | | % within row |  | 32.1 % |  | 67.9 % |  | 100.0 % |  |
|  | | % within column |  | 64.3 % |  | 73.1 % |  | 70.0 % |  |
| Total |  | Observed |  | 14 |  | 26 |  | 40 |  |
|  | | % within row |  | 35.0 % |  | 65.0 % |  | 100.0 % |  |
|  | | % within column |  | 100.0 % |  | 100.0 % |  | 100.0 % |  |
|  | | | | | | | | | |

| χ² Tests | | | | | | | |
| --- | --- | --- | --- | --- | --- | --- | --- |
|  | | **Value** | | **df** | | **p** | |
| χ² |  | 0.335 |  | 1 |  | 0.563 |  |
| N |  | 40 |  |  | |  | |
|  | | | | | | | |
